# The Academic Freedom Index and Its indicators: Introduction to new global time-series V-Dem data

**DOI:** 10.1007/s11135-022-01544-0

**Published:** 2022-10-13

**Authors:** Janika Spannagel, Katrin Kinzelbach

**Affiliations:** 1 SCRIPTS, Freie Universität Berlin, Berlin, Germany; 2 Institute of Political Science, FAU Erlangen-Nürnberg, Erlangen, Germany

**Keywords:** Academic freedom, Institutional autonomy, Right to science, Human rights, Expert assessments, Dataset

## Abstract

The *Academic Freedom Index* is the first conceptually thorough assessment of academic freedom worldwide and a times series dataset going back to 1900. While some previous datasets exist, they are geographically limited and methodologically or conceptually insufficient to offer a comprehensive picture of the levels of academic freedom across time and space. This paper introduces the new expert-coded dataset that includes the overall *Academic Freedom Index* alongside several specific indicators, to which more than 2050 country experts around the world have contributed and which is freely available as part of V-Dem’s time-series data releases. The paper discusses its advantages compared to other types of data on academic freedom, details the conceptualization of the new indicators, and offers a content and convergent validation of the results. The dataset provides ample opportunities for scholars to conduct in-depth research on academic freedom and its infringements, and for policymakers and advocates to monitor and analyze patterns and trends of academic freedom around the world.

## Introduction

The freedom indispensable for scientific research is recognized as a human right and has been widely endorsed by states parties to the International Covenant on Economic, Social and Cultural Rights. From incident-based reporting, we know that academic freedom is routinely violated across the world.^1^ Due to the lack of an appropriate comparative measurement, however, our broader understanding of global levels of academic freedom has been very limited up until now. In a collaborative effort we developed new expert-coded and factual indicators to close this gap.^2^ They are part of V-Dem’s survey on Civic and Academic Space, freely available online and regularly updated as part of V-Dem’s annual data release (V-Dem 2022).^3^ More than 2,050 country experts around the world have contributed to the data collection of the AFI.

The present paper introduces the dataset, consisting of one pre-existing and seven new V-Dem indicators, of which five are aggregated into a new *Academic Freedom Index* (AFI). In its latest release the index is available from 1900 to 2021 and covers all 179 contemporary countries or territories currently coded by V-Dem. The data are updated annually, and we aim to close the few remaining gaps in the coming releases.^4^ In this paper, we first discuss the benefits of our assessment approach in comparison to previous attempts to measure academic freedom and explain the underlying concept and operationalization of our indicators. In Sect.  3, we introduce all indicators in greater detail, giving insights into the rationales behind certain coding and wording decisions. In Sect. 4, we offer a content and convergent validation of the *Academic Freedom Index* and its indicators following the framework of Adcock and Collier (2001).

## Academic freedom

### Existing datasets relating to academic freedom

Some comparative assessments of academic freedom already exist, which rely on five different types of data: events-based data, self-reporting data, legal analyses, survey data, and expert-coded data. However, these methods or their implementation so far are insufficient to paint a comprehensive picture of academic freedom violations across time and space.^5^ In fact, none of the existing data sources’ coverage expands beyond recent years and most are geographically very limited in scope.

Events-based data on attacks against academics and students have been collected by Scholars at Risk’s Academic Freedom Monitoring Project since 2013 (SAR n.d.). Other examples include the dataset by the Global Coalition to Protect Education from Attack (GCPEA 2020) or by national-level initiatives such as the Egyptian Association for Freedom of Thought and Expression (AFTE 2017). While such data are a very useful tool to illustrate and showcase academic freedom violations, research on conflict and human rights data shows that events-based data have critical limitations when it comes to depicting violence and repression (Price and Ball 2014). Their exclusive focus on events of repression or violence means that existing institutional restrictions and systematic intimidation remain unreported. One paradoxical effect of this omission is that such data can make the most repressive environments appear comparatively benign. Furthermore, events data collected at the international level are known to be selective and heavily biased in favor of certain types of events against others, in particular violent ones. Therefore, they do not allow for a representative description of a given phenomenon. In addition, the number of known and reported events are crucially dependent on the overall availability of information and the capacity of observer organizations. These are likely to fluctuate over time, so that event counts wrongfully suggest that they depict a change in repression levels – when in reality they show changing levels of information (Clark and Sikkink 2013) and data collection capacity (Spannagel 2019).

Institutional self-reporting data are, for example, at the core of the European University Association’s Autonomy Scorecard, which assesses the autonomy of universities across Europe (EUA 2017).^6^ A similar approach is taken by the Times Higher Education (THE) ranking in its relatively new University Impact Ranking, aiming to measure universities’ contribution towards the Sustainable Development Goals (SDGs). Amidst a broad range of other indicators, THE includes the existence of “policies guaranteeing academic freedom" as part of its assessment of SDG 16: Peace, Justice and Strong Institutions. Their data are collected exclusively through self-reporting and on a voluntary and selective basis (THE 2020). Like events-based data, self-reporting data have fundamental limitations when it comes to obtaining a comprehensive global measure of academic freedom—they can cover only relatively factual questions and are easily subject to manipulation, especially in closed political environments where it is difficult to independently verify the information submitted.

In scholarly work, various authors have focused on comparative analyses of legal guarantees for academic freedom or for specific aspects such as institutional autonomy or tenure for academics (e.g. Karran et al. 2017; Appiagyei-Atua et al. 2016). The main problem with a purely legal analysis at a global level is that it risks capturing a misleading picture when not compared to a country’s *de facto* situation of academic freedom, as discrepancies between law and practice are likely to be high in many countries around the world. This concern is confirmed by analyses of the new dataset: In 2021, for example, more than 75 percent of countries with the worst performances on academic freedom (i.e., AFI scores of less than 0.2) have constitutional protections for academic freedom in place (Spannagel 2022).

Another frequent approach is to work with survey-based data collections on academics’ perception of their academic freedom, as done, for example, by Karran and Millinson (2018) in the UK and European context, by the Institut für Demoskopie Allensbach in the German context (Petersen 2020), or among China-focused social scientists outside of mainland China by Greitens and Truex (2019). The insights gained through such data can be valuable, but are at the same time limited both in their substance and geographic application. Representative survey data cannot appropriately be collected at a global level on account of the ethical concerns associated with surveys in authoritarian contexts as well as the likely self-selection, censorship and manipulation of participants and responses.

In determining our own measurement approach, we carefully considered different options, consulted with a range of stakeholders (cf. Hoffmann and Kinzelbach 2018), and found that expert-coded data would be the most appropriate way to reliably evaluate academic freedom at a global level. The consultation of experts is a very valuable tool to obtain comprehensive, informed assessments of a given issue in a given country. Experts can contextualize and integrate various types of information, and thus also overcome data gaps that exist in other sources. These characteristics make expert-coded data particularly convenient for systematic evaluations of an issue like academic freedom over time and across countries. For this reason, expert-based indices are already well-established in the field of democracy and political freedom measurement, including the Varieties of Democracy (V-Dem) Project, Freedom House or the Bertelsmann Transformation Index. There are also downsides to the use of expert assessments, however. The biggest obstacles to generating good expert-coded data are the difficulties to find enough qualified and reliable experts, and to cross-validate, aggregate and calibrate various individuals’ assessments in a way that produces balanced and comparable results across units and time (cf. Spannagel 2020). The V-Dem Project, which implemented the data collection for the new academic freedom indicators, addresses these challenges in their methodological approach (see more in Section 3.2 below).

Before we established the new indicators on academic space as part of the V-Dem dataset, none of the global democracy datasets using experts assessments had included a meaningful measure of academic freedom. Only Freedom House and V-Dem addressed academic freedom at all,^7^ but their existing indicators were conceptually insufficient to assess the concept in its complexity. Freedom House’s indicator D3 (“Is there academic freedom, and is the educational system free from extensive political indoctrination?") focuses mainly on political expression, and relates not only to higher education, but also to primary and secondary education. What is more, the indicator does not specifically address the freedom to research, but only that of teaching (see the D3 sub-questions at Freedom House 2022). V-Dem already had one pre-existing indicator related to academic freedom in their dataset, v2clacfree (“Is there academic freedom and freedom of cultural expression related to political issues?"), which similarly focuses exclusively on the ability to express political views while not addressing academics’ ability to *work* freely (and further fails to disaggregate the cultural and academic spheres).

Yet academic freedom and freedom of expression are distinct concepts.^8^ Important dimensions of academic freedom—such as the freedom to research and teach, institutional autonomy, and the freedom to exchange research findings with other scholars—cannot be subsumed under freedom of expression. Therefore, neither of the two existing expert-coded indicators can be regarded as meaningful measures of academic freedom. Due to these shortcomings of the existing data, the formulation of new indicators was necessary to collect data that are both more focused on the specific issue of academic freedom *and* more comprehensive in its conceptualization, and that furthermore overcome the existing critical limitations in terms of geographic and temporal coverage.

### Operationalizing academic freedom

Although there has been a rich and lengthy discussion in scientific literature about the definition of academic freedom,^9^ it is subject to contestations like any international norm (Özmen 2021). In international law, academic freedom is addressed under the umbrella of the right to science in Article 15 of the International Covenant on Economic, Social and Cultural Rights of 1966, which states in paragraph 15.3: “The States Parties to the present Covenant undertake to respect the freedom indispensable for scientific research and creative activity." This treaty is legally binding for all current 171 states parties. In April 2020, the UN Committee on Economic, Social and Cultural Rights issued a general comment on Article 15, defining this freedom as including

“At the least, the following dimensions: protection of researchers from undue influence on their independent judgment; the possibility for researchers to set up autonomous research institutions and to define the aims and objectives of the research and the methods to be adopted; the freedom of researchers to freely and openly question the ethical value of certain projects and the right to withdraw from those projects if their conscience so dictates; the freedom of researchers to cooperate with other researchers, both nationally and internationally; and the sharing of scientific data and analysis with policymakers, and with the public wherever possible" (CESCR 2020).

In our approach to assessing academic freedom we decided in favor of a universalistic angle based on international law that seeks to do justice to the multi-faceted nature of the concept. At the same time, the operationalization allows for the disaggregation of indicators that would give users of the data the option to consider different dimensions separately.

In the process of developing a systematic concept, we identified a range of elements often considered essential to the *de facto* realization of academic freedom based on a review of the literature and in-depth discussions with transnational policymakers, academics and advocates in the higher education field.^10^ Subsequently, we captured these elements with four new key indicators, which are well aligned with the CESCR’s above-quoted definition – namely the *freedom to research and teach*, the *freedom of academic exchange and dissemination*, the *institutional autonomy* of higher education institutions, and *campus integrity*^11^. The inclusion of additional aspects would have been conceivable, for example an indicator capturing academics’ general job security. But when formulating those new indicators we decided in favor of the most parsimonious solution by focusing on elements that are a) *comparable* across different university systems around the world and b) *specific* to the academic sector, i.e. that are not yet measured by existing indicators and that describe the situation distinctly from overlapping phenomena of repression and infringements of broader rights such as non-discrimination or freedom of expression. However, for the aggregate *Academic Freedom Index* we decided to complement these four measures with the indicator on *freedom of academic and cultural expression* that was already included in the V-Dem dataset. This decision will be further explained below.

Moreover, we found it useful to include some additional aspects in the dataset (not the index), namely factual information on *de jure* commitments to academic freedom.

## The new indicators on academic freedom and academic space

In this section, we present all indicators relating to academic space in more detail to give additional context, provide methodological information, and explain some of the rationales on which coding and wording decisions were based. We will start with a factual variable used to pre-code the dataset, followed by the academic freedom indicators and AFI, as well as the complementary factual indicators.

The V-Dem v12 Codebook (Coppedge et al. 2022a) should be used to complement this section, as it lists all discussed indicators including their exact wording, clarifications, response levels, source information (in the case of factual data), as well as definitions and instructions given to the expert coders. The Codebook also provides information on the different available versions of the expert-coded variables and the confidence intervals of the measurement estimates.

### Pre-coding variable: Existence of universities (factual data)

Academic freedom can only be meaningfully assessed where academic institutions exist. Therefore, for our expert-coded academic freedom indicators,^12^ we pre-coded V-Dem’s contemporary dataset, which covers a total of 183 countries and territories since 1900 (179 of which still exist today), in order to limit the academic freedom coding to those country-years where academic institutions were—or had previously been—present. Given that the closure of previously existing academic institutions could be the result or instrument of a crackdown on academic freedom, we only filtered out country-years prior to the foundation of a country’s first university or other higher education institution.^13^ For all country-years in the contemporary V-Dem dataset, the relevant v2cauni variable thus indicates whether universities currently exist or have ever existed in that country (1) or not (0). Expert coders for the four new academic freedom indicators accordingly only assess the country-years coded as (1).

The factual pre-coding data on the existence of higher education institutions were collected based on information from two main sources: the International Association of Universities’ (IAU) World Higher Education Database (WHED, whed.net) and uniRank’s website 4icu.org, an international higher education directory.^14^ Both sources list higher education institutions operating in countries around the world and provide, among other information, their foundation date. Knowing that neither source is exhaustive or necessarily accurate, we compiled a list with the oldest higher education institution of each country and compared the foundation dates between the two sources. For those countries where WHED and uniRank provided conflicting information and at least one of the suggested dates was later than 1900, we additionally consulted country-specific online sources and academic literature.^15^ In cases where these third sources did not provide evidence allowing us to clearly determine the country’s first higher education institution’s foundation date, we decided in favor of the earliest suggested date. For historic and smaller countries not included in uniRank and WHED, we equally relied on information from third sources. To address any remaining coding errors,^16^ we further asked V-Dem’s country experts who coded the academic space indicators to verify the accuracy of the coding start determined by our pre-coding dataset. For the v12 data release, we adjusted the underlying v2cauni dataset with minor corrections based on the comments provided by the experts of the v10 and v11 releases. Another round of small adjustments is planned for v13.

For years in which all of a country’s universities were closed down, coders were asked to assess the closure’s impact on the academic space in terms of academic freedom (which may differ depending on the reason for closure—the COVID-19 pandemic provides a good example of closures that were largely not politically motivated). In addition, we requested that coders add a note on such closures in a comments field.

### The Academic Freedom Index and composing indicators (expert-coded)

Assessing abstract concepts such as “academic freedom” is not a simple task, as we have highlighted above. Among the strengths of V-Dem’s approach to expert-based assessments is full transparency on the number of coders, the raw data of individual submissions, and the data aggregation procedures. V-Dem’s innovative statistical methodology takes into account coders’ potential biases, diverging coding behaviors and levels of confidence. For each expert-coded indicator, V-Dem gathers data from multiple, independent coders to provide reliable estimates. More than 2050 experts—typically academics, both in- and outside the respective country—have so far contributed assessments to the the academic freedom indicators. Country-year data points that did not meet a threshold of at least three coders were omitted from the public dataset, while aggregated index data is provided for country-years with at least three out of five indicators meeting that threshold, which is why some countries or territories do not yet have full temporal coverage in the v12 edition. As partly different pools of experts contribute to the two V-Dem surveys in which the five indicators are included, each single aggregate country-year index data point is currently based on an average of 10 independent experts.

The geographic comparability of the data is improved through bridge coders, who provide assessments for more than one country, as well as through the use of so-called anchoring vignettes, which are brief descriptions of fictitious scenarios for each indicator. When expert coders of different countries rate those hypothetical cases, it helps to estimate experts’ individual assessment thresholds on the pre-defined scale for each indicator. To aggregate the ratings of individual coders into country-year scores for each indicator (and in a second step for the index), V-Dem relies on a custom-built Bayesian measurement model with integrated inter-coder reliability tests. The model relies on item-response theory and provides the respective best estimate of the value for an observation, alongside an estimate of uncertainty for each data point. For detailed insight into the methodology of the expert-coded data and the statistical modeling behind the various estimates, see Pemstein et al. (2019).

The new *Academic Freedom Index* consists of five indicators on academic freedom, each of which is coded by country experts on a predefined scale from 0 to 4 and on a country-year basis:

- freedom to research and teach (v2cafres);
- freedom of academic exchange and dissemination (v2cafexch);
- institutional autonomy of universities (v2cainsaut);
- campus integrity (v2casurv);
- freedom of academic and cultural expression (v2clacfree).

First of all, it should be noted that we consider all undue interference by non-academic actors as infringements on academic freedom. Non-academic actors include individuals and groups such as politicians, party secretaries, externally appointed university management, businesses, foundations, other private funders, religious groups, and advocacy groups. As a consequence, we do not consider restrictions that are set *by the academic community* itself as interference, including issues regarding research priorities, ethical and quality standards in research and publication, or standardized curricula aiming to enhance teaching.

Secondly, we realize that levels of academic freedom may vary substantially between institutions and geographic regions within the same country, and acknowledge that it is a significant limitation of the new dataset that such within-country variations cannot be adequately depicted. For the country-level assessment, we asked the expert coders to generalize across universities in a particular country, while considering the prevailing practices and conditions and bearing in mind different institutions’ relative importance in the higher education sector of the country. The indicators themselves are formulated in a way that would in principle allow, in a separate data collection effort, for researchers to apply them at the institutional level. Furthermore, we recommend that the quantified country-year scores be complemented by qualitative case study information, including to explore within-country variations.^17^

A third important issue in the assessment of academic freedom infringements in a country is the likely variation between disciplines, such as between natural sciences and social sciences, established fields of study and contested ones, or economically profitable and non-profitable sectors. Under authoritarian conditions, we presume that the social sciences are, typically, under stricter control by the state. In contrast, financially profitable sciences are likely more exposed to the influence of corporate money. However, we are aiming to assess the integrity of the academic community as a whole, and consider it to be dangerous to excuse or relativize the infringements on some subjects by the freedom of others—precisely because the targeting of a few sensitive subject areas is a known pattern of repression, and often spreads a culture of fear throughout the academic community. What is more, infringements on institutional autonomy affect all academics, regardless of their discipline. At the same time, the quality of restrictions on the academic sector as a whole is different depending on whether only some or all disciplines are targeted, which is why we didn’t choose to focus on only the worst-off subject areas. We instead decided to include a qualification on the scope of infringements across disciplines in the response scale of the first two indicators (the freedom to research and teach and the freedom of academic exchange and dissemination). Accordingly, countries are rated as “completely restricted" (0) if interference and restrictions are consistently applied *across all disciplines*, and as “severely restricted" (1) if *in some disciplines* interference and restrictions are consistently applied. However, the frequency with which interference and restrictions occur also matters greatly in determining the level of academic freedom. The remaining levels “moderately restricted" (2), “mostly free" (3) and “fully free" (4) are therefore defined by that frequency (occasionally, rarely, or not, respectively) instead of by the scope across disciplines (for more details see Coppedge and al. 2022a).

Regarding the second indicator on the freedom of academic exchange and dissemination, we consciously decided to include both so-called intramural and extramural freedoms in the same indicator. The terms refer to the freedom to discuss and disseminate research findings among academic (intramural) and non-academic audiences (extramural). The rationale of this decision was that we consider them to be inseparable elements of the broader issue of academic exchange and dissemination. If considered separately, countries that systematically restrict extramural but not intramural exchanges (e.g. allow the publication of research results only in highly technical, scholarly journals) would likely end up somewhere in the middle, instead of low, on this measure. Such operationalization would problematically suggest that academic freedom may be legitimately restricted to the intramural sphere. Our decision is in line with the interpretation of the UN Committee on Economic, Social and Cultural Rights, which has expressly recognized that scholars may share scientic data and analysis with policymakers and with the public (CESCR 2020).

The third indicator assesses the extent to which universities exercise institutional autonomy in practice. Here, it should be noted that autonomy does not preclude universities from accepting state or third party funding and from being accountable towards that funder, but does require that the university remains in charge of decisions regarding its internal governance, finance, administration, research choices and findings, methods to be applied, and similar issues.

Our fourth indicator assesses what we call *campus integrity*, where we understand by “campus" all university buildings as well as digital research and teaching platforms. Campus integrity means the preservation of an open learning and research environment marked by an absence of a deliberately, externally induced climate of insecurity or intimidation on campus. Examples of infringements of campus integrity are politically motivated on-campus or digital surveillance, presence of intelligence or security forces, presence of student informants or militias, and violent attacks by third parties when specifically targeting universities to repress academic life on campus. What is important to note for this indicator is that we are only interested in targeted attacks on campus integrity – and not in security concerns or proportionate security measures taken on campus to address them. This is a crucial distinction, as the assessment of some measures such as CCTV cameras on campus requires contextualization: While in one context they may aim to provide security against external threats or facilitate long-distance learning, they can serve as instrument of intimidation and control in other situations. Similarly, the location of a university campus in a generally insecure conflict zone should not automatically result in a lower score on campus integrity, unless targeted attacks disrupt or influence academic work.

Lastly, we decided to rely on V-Dem’s pre-existing measure of *freedom of academic and cultural expression* to complete the *Academic Freedom Index*.^18^ Although its formulation—including the amalgamation of academic areas with cultural work—is not ideal, it is a valuable indicator that captures academics’ freedom of expression in relation to political issues. As mentioned above, we consider academic freedom and freedom of expression as distinct concepts, but we acknowledge that they can overlap and that the line is often difficult to draw, which is why we decided to include this measure in the index. The fifth indicator’s inclusion also has advantages from a methodological point of view, as it is a measure that has been in the V-Dem dataset for several years and is included in a different survey than the indicators on academic space. Therefore, it is not only a well-established indicator with often high coder numbers, but its assessment can also be regarded as largely independent from the other four measures—thus improving the objectivity of the composite index measure.

The *Academic Freedom Index* is obtained through aggregation by point estimates drawn from a Bayesian factor analysis model (cf. Coppedge et al. 2022b). Its range of values is between 0 and 1. We believe that this aggregation and our conceptualization provide a meaningful overall assessment of academic freedom. We acknowledge, however, that there are other possible ways of combining the newly available data into an aggregate measure. Different compositions, weights and aggregation methods may therefore be applied by individual researchers.

### Complementary measures: *De jure* commitments (factual data)

Next to the indicators on the *de facto* realization of academic freedom, two new factual variables were released that measure different elements of states’ *de jure* commitment to academic freedom. The first provides information on whether constitutional provisions for the protection of academic freedom exist (v2caprotac) and the second captures states’ international legal commitment to academic freedom under the International Covenant on Economic, Social and Cultural Rights (ICESCR, v2caacadfree). Together with the expert-coded indicators of *de facto* levels of academic freedom, these variables allow us to compare divergence and convergence of *de jure* protections and *de facto* academic freedom across time and space. In addition, the ICESCR data enhance research opportunities into the effects of international human rights treaties on national *de jure* and *de facto* implementation.

The data on constitutional provisions were contributed by the Comparative Constitutions Project (CCP, Elkins et al. (2020) and cover country-years since 1900. The coding distinguishes between no provision (0), existing provision (1), constitution suspended in that year (95), other or undetermined phrases (97), and a code for missing data (99). Cells are left blank for country-years that are not applicable, i.e. mainly in the case of non-sovereign territories.

In line with the CCP methodology, constitutional texts were typically examined by two independent coders searching the constitutions for a guarantee of academic freedom. In the event that two or more coders disagreed with each other, the answers were reconciled by a third, more experienced person who adjudicates competing answers. A small number of cases was single-coded, which are indicated by a confidence level of 0.75 (compared to 0.95 in other cases).^19^ In terms of content, it is important to highlight that CCP applied a relatively broad understanding of “academic freedom provision," which includes, for example, references to the freedom of education.^20^ An improved version of this dataset on constitutional academic freedom codifications, using a narrower and more consistent definition and providing more comprehensive coverage, is currently in the making (cf. Spannagel 2022) and will replace the original v2caprotac variable from v13 onward.

The second variable, on states’ international legal commitment, was put together by Janika Spannagel and Alicja Polakiewicz. It indicates whether the country is party to the covenant without having made explicit reservations to its Article 15 (right to science), which stipulates, among other things, that states parties “undertake to respect the freedom indispensable for scientific research". The indicator covers the years since 1966, when the treaty was adopted and opened for signature; only states with UN member status or UN non-member observer status that allows treaty participation are coded.^21^ Note that we coded the ratification status as of December 31st of each year.

The coding distinguishes between the following: (0) State not a party to ICESCR, or made reservations to Article 15; (1) State is party to ICESCR without reservations to Article 15, but treaty not yet in force; (2) ICESCR in force and signed without reservations to Article 15; (3) ICESCR in force and ratified without reservations to Article 15. Cells are left blank for country-years for which the indicator is not applicable, i.e. states or jurisdictions with neither UN member nor observer status.^22^

It should be noted that, although the language of the coding suggests otherwise, we found that no state has so far made any explicit reservation to Article 15. Nevertheless, we decided to leave the indicator’s wording unchanged to highlight states parties’ specific—and unqualified—commitment to the right to science and, thereby, academic freedom (and also because states may in the future decide to file reservations on Article 15).

Regarding states emerging from dismemberment or secession, we treated cases differently depending on whether the respective new state eventually declares treaty *succession* or *accession*. If a state breaks away from a previous entity that had signed or ratified the treaty and the state later declares succession to the treaty, then the predecessor state’s status is coded without interruption. If a state breaking away later declares accession to the treaty (or signs and ratifies; or declares nothing at all), then the predecessor state’s status ends with formally declared independence. The new state is not coded—i.e. blank—until it obtains UN membership, and then coded “not state party" (0) until accession is declared or the treaty is signed/ratified.

## Data validation

With the expert-coded indicators on academic space, we aim to measure abstract concepts whose true values are unknown. To review the data’s validity, we use content and convergent validation approaches (Adcock and Collier 2001). Content validation serves to evaluate whether a given indicator adequately captures the full content of a systematized concept, largely by determining whether it captures empirically relevant variation across countries and time. Convergent validation serves to investigate whether alternative indicators of a given concept are empirically associated, i.e. convergent, and thus really point to the same concept.

### Global trends of academic freedom over time

**Fig. 1:**
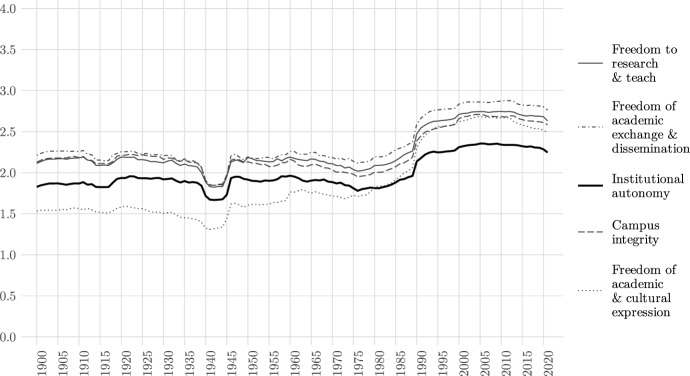
Global Trends in Indicators of Academic Freedom 1900-2021. Source: V-Dem data (v12)

Global average levels of the *Academic Freedom Index*’ five constituent indicators over time are depicted in Fig.  1. The trends are similar between the indicators—with some important exceptions. Overall we see a small dip in global levels on all academic freedom indicators during World War I (1914–1918) and a very substantial dip during World War II (1939–1945). Furthermore, all indicators show a slow degradation between the early 1960s and the late 1970s—likely associated with repressive policies in the Soviet Union, the installment of several military dictatorships in Latin America, as well as Cold War–related pressures on academia in other parts of the world. The 1980s are a period of slow improvements, which accelerate in the early 1990s with the third wave of democratization before stabilizing at a comparatively high level. Since 2013, we see a slight decline in several variables, which, however, remains within the statistical error of the data (for a more detailed analysis of recent trends, see Kinzelbach et al. 2022).

The comparison between indicators suggests that institutional autonomy is more inert than other indicators when sudden changes occur, and we can see that since 1990 it has settled at a substantially lower level on average than other indicators. Although we are measuring the *de facto* level of institutional autonomy, this is consistent with the expectation that institutional processes are slower to change than those affecting other indicators, which should be more sensitive to sudden events or their absence.

The freedom of academic and cultural expression, on the other hand, shows similar levels of fluctuation as the non-autonomy indicators. While it is consistently lower than all four indicators until the late 1970s, from this point onward its global average value rises steeply to reach similar levels as the three indicators other than institutional autonomy. It shows the clearest decline in recent years, while institutional autonomy hardly shows notable changes. Given that academics’ freedom of expression should be more sensitive to changes in a country’s political environment than the other academic freedom indicators, these patterns appear plausible.

### Country-level trends of academic freedom over time

**Fig. 2:**
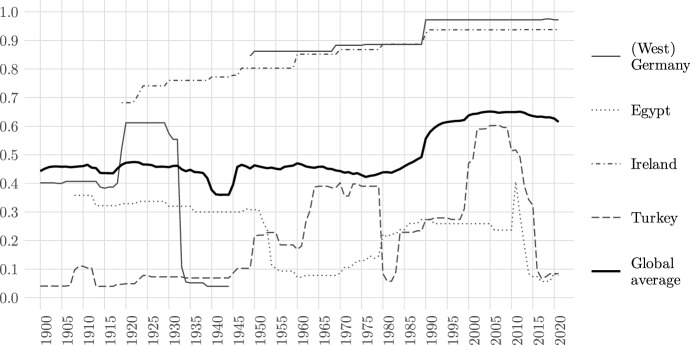
Trends in academic freedom in select countries 1900–2021 (AFI). Source: V-Dem data (v12)

In terms of country-level trends, a comparison of the *Academic Freedom Index* between select countries with strongly diverging trends in academic freedom over the covered time period (Fig. 2) reveals patterns that are consistent with empirically important periods in the respective contexts. In Germany, we see a level of academic freedom just below the global average during the time of the German Empire, which then rises above average during the Weimar Republic and drops to a near-absence of academic freedom during the Nazi regime.^23^ Post-War Germany (representing only West Germany until 1990) starts off with high levels of academic freedom and step-like improvements in the late 1960s and at the end of the Cold War, which then stabilize at a very high level. Ireland starts off with relatively high levels of academic freedom and gradual improvements over time, coinciding with factors including the gradual reduction in church influence, and improvements in formal university autonomy in the early 1970s.^24^ In contrast, Turkey’s levels of academic freedom have been more volatile over time. With the introduction of the multiparty system after 1945, academic freedom increases. The years leading up to the 1960 military coup, sometimes referred to as the “progressive coup," show lower levels of academic freedom. They increase again with the return to civilian rule. The coup in 1971 briefly has a negative effect on levels of academic freedom in Turkey, but it is the 1980 military coup that hit universities particularly hard, as shown by the index’ sudden drop. The steep increase to levels near the (overall also increased) global average in the early 2000s coincides with the beginning of a so-called liberal era in Turkish politics, though individual academics continued to be targeted. Since 2010, authoritarian measures have accelerated and we see levels of academic freedom drop to a level of near-absence alongside the pressures that followed the 2016 coup attempt.^25^ In Egypt, a steep decline from middle-range levels of academic freedom can be observed with Nasser’s coup in 1952, which slowly improve thereafter under Sadat (1970s) and Mubarak (1981–2011)—though remaining at a relatively low level overall. After a brief and small spike after the 2011 revolution and the first free elections, academic freedom drops to Sadat-era levels after the military coup that removed Mohamed Morsi from power, and thereafter declines to a historic low.^26^

These country graphs illustrate that the *Academic Freedom Index* captures important periods in countries’ histories. In terms of data quality, we find differences in the granularity of scores between countries and across time, however, which is determined by the attention to detail provided by individual coders.

**Fig. 3:**
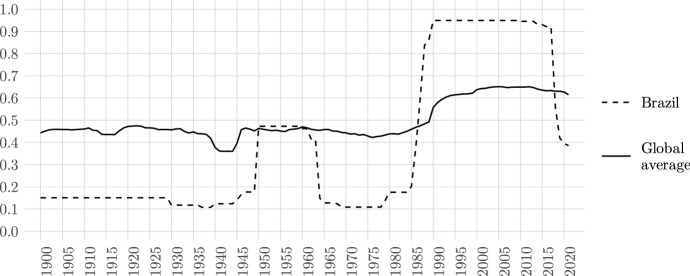
Trends in academic freedom in Brazil 1980–2021 (AFI). Source: V-Dem data (v12)

In addition, we have reason to believe that recent worrisome developments tend to be overly reflected in the data. As an illustration, Fig. 3 shows the development of the *Academic Freedom Index* in Brazil over the past four decades. While there is definitely evidence of a deteriorating condition for academics in the country (Hübner Mendes 2020), the extent of the score’s decline over the last four years seems somewhat disproportionate in comparison to earlier periods in the country’s history, as well as in comparison to other countries in the world over the same period. We believe that such tendencies are an intrinsic feature of expert-coded data that need to be acknowledged and openly discussed. As many expert contributors are themselves part of the respective country’s academic system and are currently experiencing a period of deterioration and major uncertainty, their concerns are reflected in the data. Rather than considering this as problematic per se, we believe that these trends should be read as important warning signs that depict the current climate in the country. However, we also encourage continuous critical engagement with the data, as well as additional expert assessments in future rounds of data collection that allow for a retrospect evaluation of the situation.

### Country-level variation between academic space indicators

**Fig. 4:**
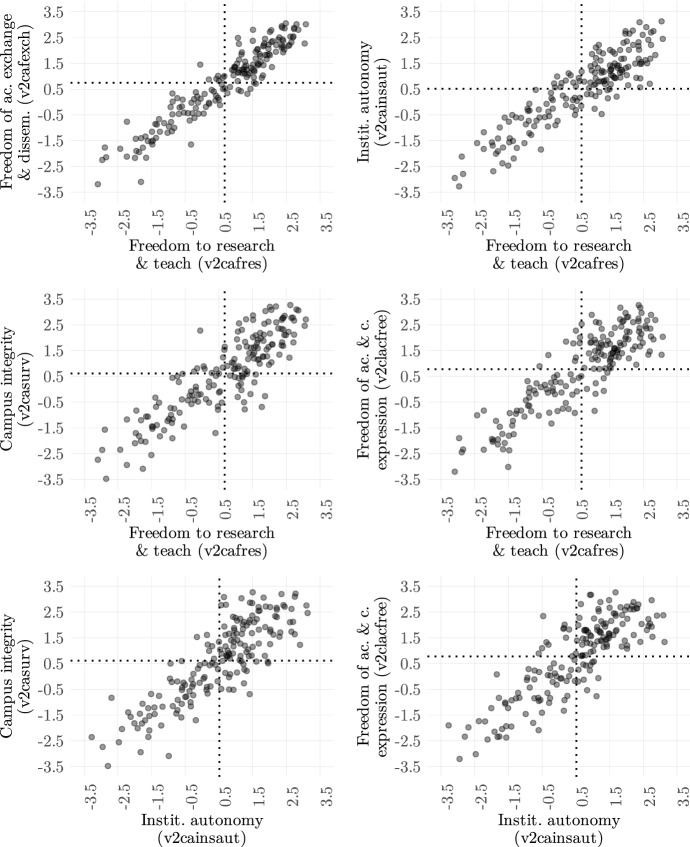
Relationship between various Academic Freedom Indicators (2021). Dotted lines represent mean values. Source: V-Dem data (v12)

The scatter plots in Fig. 4 illustrate the relationships between various pairs of the five academic freedom indicators for 2021 (dotted lines show the respective means). In the upper left plot, we find that the two indicators that are most closely associated are *freedom to research and teach* (v2cafres) and *freedom of academic exchange and dissemination* (v2cafexch). The correlation across the whole dataset (1900–2021) is very high at 0.95. Given the close association of the two concepts, this meets our expectations.

Between v2cafres and universities’ *institutional autonomy* (v2cainsaut), the indicators seem more closely associated at very low levels in 2021: in the absence of freedom to research and teach, there is typically no institutional autonomy, and vice-versa. High levels in v2cafres do, however, regularly coexist with middle-range levels in v2cainsaut. The correlation between the two is at 0.88 across all country-years, supporting the idea that institutional autonomy overall facilitates freedom to research and teach.

The relationship between *freedom to research and teach* (v2cafres) and *campus integrity* (v2casurv) is similar to that with institutional autonomy, except that at very high levels they seem to again closely associate. Since campus integrity violations tend to include relatively drastic measures of policing and surveillance, this is not surprising. On the other hand, campus integrity is not necessarily completely absent in countries with very low freedom to research and teach, as some outliers show. Since institutional autonomy is very low in those cases, campus integrity violations may not be necessary from an autocrat’s perspective to keep those universities in check. Indeed, when comparing autonomy (v2cainsaut) and campus integrity (v2casurv), we find more dispersion (correlation of 0.84 across the dataset), which would support the idea that institutional and physical control are distinct tactics of repression and may—but do not have to be—used alongside each other. The fact that the two concepts are not as closely associated is thus reflected in the data.

The *freedom of academic and cultural expression* (v2clacfree) shows a relatively consistent association with *freedom to research and teach* v2cafres (correlation of 0.84 across all country-years), in particular where both levels are high (see right plot in second row for 2021). Around the mean value of v2cafres, however, we see a large range of values in v2clacfree. The correlation of v2clacfree and *institutional autonomy* (v2cainsaut) is overall substantially lower (0.77), reflecting the less close conceptual association of universities’ institutional autonomy with academics’ freedom of expression on political issues. The generally middle-to-high correlation with the new indicators makes sense, given our expectation that higher levels of overall freedom of expression (and in particular for academics) also means greater freedom in their scientific work. The divergences, however, also support our reservation regarding the use of the v2clacfree variable as a valid exclusive measure of academic freedom—suggesting that the four new indicators provide more substance to the concept of academic freedom as a whole, as captured by the new *Academic Freedom Index*.

### Comparison With Freedom House indicator on academic freedom

By comparing the new academic freedom indicators with the *freedom of academic and cultural expression* (v2clacfree) variable, we already tested their association with a measure that is external to our own expert survey. The analysis of global trends between the five indicators supports the expectation that v2clacfree is more closely aligned with a country’s overall political environment, while showing middle-to-high correlations with the new indicators.

The only other existing global measure of academic freedom to date is provided by Freedom House. As discussed in Sect. 1.1, it also focuses on political expression and additionally includes primary and secondary education in its measurement. We thus expect that the correlation of Freedom House’s D3 measure should be highest with v2clacfree. Though this is the case (at 0.85), the correlations with v2cafres, v2cafexch and v2casurv are not too far off (0.81, 0.82 and 0.81). Institutional autonomy is correlated with Freedom House’s measure at 0.78.^27^

**Fig. 5:**
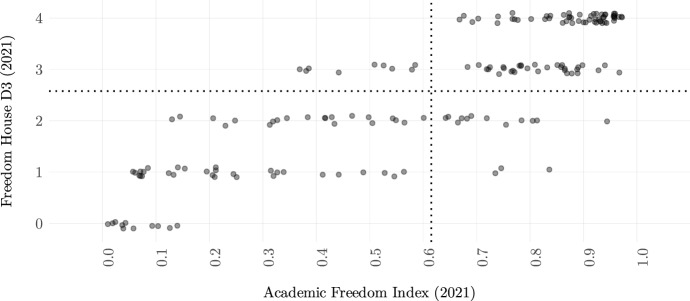
Relationship between *Academic Freedom Index* and Freedom House’s indicator on academic freedom (2021). Dotted lines represent mean values. Compared are data for 2021 from V-Dem’s v12 dataset and data for 2021 from Freedom House’s 2022 report edition. The data are jittered along the y-axis for better readability

Figure 5 illustrates the relationship between Freedom House’s indicator and the *Academic Freedom Index* for 2021. Although we see that there is overall a clear relationship (correlation at 0.85 for country-years since 2012), many countries are only roughly located at the same level between the two indicators. Assessments vary more in the middle of the scale than on the extremes. In some cases, the divergences may be an artifact of the inclusion of primary and secondary education in Freedom House’s measure, as well as its exclusive focus on political expression. However, a cursory review of the relevant country scores reveals that these different scores cannot be explained by definitional questions alone. Further research is therefore needed to assess and reduce this relative uncertainty over the true levels of academic freedom in countries ranked in the middle of the scale.

## Conclusion

Academic freedom is fundamental for scientific progress, the pursuit of truth, research collaboration, and quality higher education. Despite many self-commitments by universities and states to safeguard academic freedom, it is under attack in many places. The lack of adequate data has so far prevented researchers from studying the phenomenon more systematically at a global scale, and impeded efforts by policymakers and advocates to monitor and act on such violations.

The new dataset introduced in this paper includes eight indicators related to academic freedom and academic space, as well as one aggregate *Academic Freedom Index*. It is the first such dataset on the topic that provides near-global and longitudinal coverage. Building on V-Dem’s well-established expert surveys and measurement model, this dataset thus represents a major new resource of conceptually sound and empirically valid measures of academic freedom and its different dimensions. A key concern with the few previously existing assessments was that they tended to equate academics’ free expression with academic freedom. The convergent validation of the new data underscores that measures of free expression are not sufficient to capture academic freedom in a comprehensive manner. The *Academic Freedom Index* and its indicators will thus help to close the existing knowledge gap and facilitate action to defend academic freedom around the world.^28^ An overview and discussion of recent trends visible in the data can be found in our annual update (Kinzelbach et al. 2022).

We believe that research into the topic of academic freedom can be significantly expanded on the basis of the new data and are encouraged to see that scientific studies that rely on the AFI data are already emerging.^29^ Possible further research avenues could, for example, be to examine the determinants and effects of shifting levels of academic freedom, the relationship between the different dimensions of academic freedom over time or an analysis of the association between academic freedom and the quality of higher education—or, for that matter, between academic freedom and freedom of expression. As mentioned above, an important omission in the data remains the within-country variation. Thus, we also encourage scholars to contribute more country case studies on the topic, for which we have developed research guidelines and published sample studies in Kinzelbach (2020) and Roberts Lyer et al. (forthcoming). Moreover, while the AFI data collection and aggregation follows rigorous scientific procedures, we acknowledge that expert assessments have inherent limitations, and welcome continuous and substantiated critical engagement with the data. In the same vein, we appeal to country experts and higher education experts to participate in future rounds of V-Dem’s data collection on the topic to help fill remaining gaps, capture nuances between years and consolidate the dataset.

## Footnotes

1 For example, Scholars at Risk’s (SAR) Academic Freedom Monitoring Project has reported over 1000 verified incidents of attacks against higher education personnel around the world between 2013 and 2020 (SAR n.d.).

2 In addition to Staffan I. Lindberg and Anna Lührmann (V-Dem Institute), Robert Quinn (Scholars at Risk) and Ilyas Saliba (Global Public Policy Institute) deserve special mention for their contributions during the conceptualization phase. The institutional affiliations of Anna Lührmann and Ilyas Saliba have since changed.

3 V-Dem is one of the largest-ever social science data collection efforts; its database contains more than 450 indicators and indices. Its current v12 dataset version covers 202 countries from 1789–2021 and is updated annually. V-Dem received the Lijphart/Przeworski/Verba Dataset Award 2016.

4 Only about two percent of all country-years are currently still missing, in addition to some historic countries.

5 For a more detailed discussion of the advantages and drawbacks of each of these data sources, see Spannagel 2020.

6 While having the drawback of limited geographical coverage, the EUA’s approach and focus allows for a more fine-grained analysis of university autonomy than the AFI’s global assessment can provide.

7 There are some geographically and temporally limited studies using expert-coded data to assess academic freedom, such as De Boer et al. (2010) for 33 European countries’ higher education systems between 1995 and 2008 and Anderson and Johnson (1998) for 20 countries in different parts of the world in the mid-1990s, both focusing on institutional autonomy.

8 We do, however, recognize that the two are related – which is why we decided to include the v2clacfree indicator in our aggregated index (see below).

9 See for example Fuchs (1963) or Eisenberg (1988) for a definition from the US-American perspective dominating the literature. Alternatively, there are definitions that conceptualize academic freedom not as a universal principle, but in relation to its application in a specific environment, such as in Marginson (2014). A definition focusing on the social responsibility of professors can be found in Appiagyei-Atua et al.(2017). A definition of academic freedom emphasizing institutional autonomy is provided by Altbach (2001).

10 In addition to some of the academic works listed above, a recent paper by Beiter (2019) was particularly helpful, as was a study by Roberts Lyer and Suba (2019).

11 As will be further elaborated below, by ‘campus integrity’ we understand the absence of a deliberately, externally induced climate of insecurity or intimidation on campus.

12 Note that the pre-coding applies only to the four new expert-coded indicators, not V-Dem’s pre-existing indicator v2clacfree. The pre-coding also applies to one additional expert-coded indicator included in the academic space survey (but not in the AFI), v2cacritic, which assesses the extent of scholars’ and students’ criticism of government policies.

13 By ‘universities,’ for the purpose of this dataset, we understand all higher education institutions, both public and private. They include institutions such as research universities, universities of applied sciences, undergraduate colleges, polytechnic universities, and international campuses.

14 The uniRank dataset was kindly provided to us by Brendan Apfeld, University of Texas. A similar version of it is available in the V-Dem datsaet as variable v2canuni, recording the total number of universities in a given country-year. This latter dataset version does, however, only include institutions that were listed by uniRank as degree-granting—an attribute we found to be incomplete and transnationally inconsistent. To avoid false negatives, we therefore worked with a pre-filter version that Brendan Apfeld shared with us.

15 The relevant data was jointly collected by Janika Spannagel and Alicja Polakiewicz.

16 A problem with both sources relates to the fact that they only list information on universities that still exist today, meaning that institutions that were closed or absorbed by others are not included. However, this turned out not to be a pervasive issue for the purposes of our coding.

17 For research guidelines and sample case studies see Kinzelbach (Kinzelbach (2020)). Additional country studies will soon be published as part of a new book by Roberts Lyer et al. (forthcoming).

18 We thank Anna Lührmann for her suggestion to combine the new indicators with the existing one.

19 In addition to this basic confidence level, CCP estimates the reliability of their data using a more complex model (see Melton et al. 2013). It may be interesting to note here that the reliability of the academic freedom variable is slightly lower (at 75%) than the average reliability across all variables of their dataset (including more than 600 questions about the content of constitutional systems). According to CCP, this higher discrepancy among coders indicates that the interpretation of the line between the provision of the right and the lack thereof proved to be uncharacteristically complex and multidimensional.

20 CCP operates a message board, which team members use to deliberate and reach consensus regarding cases and issues that are not self-interpreting from their coding manual. Using this process, the following phrases were determined to qualify for “Yes" (1) coding: “freedom to impart knowledge and information", “right to freely engage in teaching", “freedom of creative, scientific and technical work", “exercise of art and scientific research are free", “liberty of teaching", “instruction is free", “educational freedom", “freedom of education under state supervision"; and for “Other" (97) coding: “neutrality in teaching", “right to learn/teach", “inviolability of places of learning", “freedom of intellectual creativity". Note that this list only serves to give a broad understanding of the coding approach and is not a complete enumeration of terms included in either category, given that these are only the phrases that were considered contentious.

21 The only exception to this rule applies to Hong Kong since 1997, which is coded as “ratified" (3) because of the special agreement in the handover treaty between Great Britain and China. Furthermore, the V-Dem dataset distinguishes between Palestine/Gaza and Palestine/West Bank, which as the State of Palestine share UN observer status since 2012 and accessed the ICESCR in 2014; they were both coded accordingly.

22 States and jurisdictions coded as blank include: People’s Republic of China up until 1970, Taiwan (Republic of China) since 1971, Hong Kong up until 1996, Kosovo since 1999, former USSR states between independence and UN recognition, Somaliland and Zanzibar. These coding decisions were based on an assessment of whether or not the respective territory was in a position to officially sign or ratify the covenant in its own right.

23 During the occupation period 1945–49, Germany is not coded in the V-Dem dataset. See V-Dem’s country coding units in Coppedge et al. (2022c).

24 We thank Kirsten Roberts Lyer for sharing her assessment on Ireland with us. See also Roberts Lyer and Potapova (2020)’s contemporary case study on Ireland.

25 We thank Olga Selin Hünler for sharing her assessment on Turkey with us. See also her case study on Turkey (Hünler forthcoming).

26 We thank Ilyas Saliba for sharing his assessment on Egypt with us. See also his contemporary case study on Egypt in Saliba  (2020).

27 Correlations are calculated across all country-years for which Freedom House data on indicator D3 is available (2012–2021).

28 We discuss the potential use of the data by multiple stakeholders in the higher education field in Kinzelbach et al. (2021a) and the general role of academic freedom data in higher education governance in Kinzelbach et al. (2021b).

29 See for example Kratou and Laakso (2022), Berggren and Bjørnskov (2022a, b) and Kinzelbach et al. (2021b).
